# Comparison of Surgical Conditions During Propofol or Isoflurane Anesthesia for Endoscopic Sinus Surgery

**DOI:** 10.5812/aapm.9891

**Published:** 2013-09-01

**Authors:** Shideh Marzban, Soudabeh Haddadi, Hossein Mahmoudi Nia, Abtin Heidarzadeh, Shadman Nemati, Bahram Naderi Nabi

**Affiliations:** 1 Anesthesia Research Center, Guilan University of Medical Sciences, Rasht, Iran

**Keywords:** Anesthesia, General, Propofol, Endoscopy, Hemorrhage

## Abstract

**Background:**

The Anesthesia planning is an important and determining factor in the amount of intraoperative hemorrhage, which can affect the rate of intraoperative and postoperative complications.

**Objectives:**

In this study we used two different anesthesia techniques in functional endoscopic sinus surgery (FESS) and compared the amount of hemorrhage in the two groups.

**Patients and Methods:**

In a single–blind clinical trial, 44 patients with ASA class I and II candidate for FESS in Amir-Al-Momenin hospital in Rasht were entered the study and divided into two equal groups randomly. In both groups anesthesia was induced with propofol, remifentanil and cis. atracurium and then, infusion of propofol – remifentanil in the first group and isoflurane plus Remifentanil infusion in the second group was used for maintenance of anesthesia. Systolic blood pressure was maintained about 90 mmHg. Then on the basis of maximum allowable blood loss (MABL) formula, we calculated the percentage of hemorrhage. Finally the patients' hemorrhage was categorized into three groups (< 10%, 10-20%, > 20%). The surgeon's satisfaction from surgical field was calculated according to the Visual Analogue Scale. Then the data was statistically analyzed with T- test.

**Results:**

There were meaningful differences between average of hemorrhage (propofol group = 155cc, and Isoflurane group = 291.3cc; P = 0.003), and surgeon’s satisfaction (propofol group = 1.9 and Isoflurane group = 2.95; P = 0.007).

**Conclusions:**

The amount of hemorrhage in propofol group was less than Isoflurane group and the field condition was better in propofol group than the Isoflurane group.

## 1. Background

Functional endoscopic sinus surgery (FESS) is a skillful surgical technique in chronic rhinosinusitis treatment (1). When FESS is performed under general anesthesia, controlled hypotension (mean arterial pressure between 60-70 mmHg) can provide better surgical field condition and decreases the time of operation. Also occurrence of important complications such as severe bleeding, defects in skull base, intraorbital bleeding and blindness decreases (1-3). During FESS, mucosal bleeding in surgical field, often interacts with surgeon’s vision of intranasal anatomy; then the time of operation increases and more complications may occur (1, 2). Many different methods have been used to control bleeding during the operation, such as local injection of vasopressors, head up position, using hypotensive drugs, and tight control of CO_2_ (1-4).

The anesthetic drugs also can affect the amount of bleeding and surgical field situation by vasodilation and reducing the blood pressure (BP). The general idea in many studies is based on that general anesthesia with propofol provides better vision of surgical field and less amount of hemorrhage during the operation compared to Isoflurane or Sevoflurane (5). A group of studies suggest that premedication with oral clonidine decreases intraoperative bleeding in some surgeries (6, 7). Metoprolol has shown to decrease bleeding in nasal sinuses (8). Propofol is one of the most common anesthetic drugs used in general anesthesia, which decreases systemic blood pressure by vasodilation (9). In maintenance phase of anesthesia, infusion of propofol reduces the blood pressure for about 20-30 percent, compared to preinduction BP (9). The inhalational anesthetics also reduce arterial blood pressure related to their concentration, but the mechanisms are different (10). In some articles and studies, different methods and routs for providing the better field of operation and surgeon’s vision and less complications during FESS are reviewed such as: Use of intravenous and inhalational anesthetic drugs, administration of beta blockers for premedication in FESS, use of vasopressors in combination with local anesthetics during the operation and the effect of reverse trendelenburg position (3-5, 11, 12).

## 2. Objectives

With reviewing the previous articles and studies, we did not find the use of remifentanil in anesthesia for FESS, and with attention to the progressive use of FESS in our country in recent years, we decided to compare the surgical conditions in anesthesia with propofol and remifentanil versus isoflurane anesthesia in endoscopic sinus and rhino surgeries in Amir-Al-Momenin academic hospital in Rasht- IRAN.

## 3. Patients and Methods

After writing the proposal and getting ethical approval, justification register number from vice-chancellor of research department of Guilan University of Medical Science registration in Iranian Randomized Clinical Trial site(IRCT) (No: IRCT 201102081138N7), we started this single-blind clinical trial study. We recruited 44 patients (22 patients for each group) according to sample formula with ASA class I and II and with ages between 15-45 years candidate for elective endoscopic sinus surgery (13) (Inclusion criteria). Exclusion criteria were as follows: Patients with history of bleeding disorders, patients under treatment with drugs which affect surgical hemostasis, anemia (Hemoglobin concentration < 10 mg/dl), convulsion, systemic hypertension, uncontrolled Cerebrovascular Accident(CVA), coronary artery disease, arrhythmia, hepatic or renal dysfunction, electrolyte or metabolic disorders, Chronic Obstructive Pulmonary Disease, severe asthma and pregnancy. Prolonged operation (more than 150 minutes), hemorrhage more than maximal allowable blood loss (MABL). After explaining the issues about the study and fulfilling the informed consent, the patients were divided into two groups randomly by using cards that labeled with P or I. The simple randomization was performed with choosing P or I cards by patients. In this study, patients were categorized based on scores reported in CT-scan according to involved paranasal sinuses before the operation. Each affected sinus got 2 scores. They were divided into low Lund Mackay (less than 12) and high Lund Mackay score (more than 12 scores) (13). Lund Mackay score is widely used in assessment of chronic rhinosinusitis. This scoring system consists of a scale of 0-2 dependent on the absence, partial or complete opacification of the sinus system in CT scan. The scoring system derives a maximum score of 12 per side). For calculating the amount of bleeding through the operation we subtracted the amount of irrigating solution from total amount of fluids in suction bottle. Then patients were divided into three groups based on the F rate of patient's blood loss to the maximal allowable blood loss (MABL) (14): less than 10 percent, between 10 and 20 percent , more than 20 percent.

MABL = Hb _start_-Hb _target _÷ Hb _start_ × EBV

All of the patients with chronic sinusitis and with minimally two paranasal sinuses involvement were included in our study. Then the patients were divided into two groups: 22 patients in (P) propofol group and 22 patients in (I) isoflurane group. Before starting the operation, paranasal sinuses and CT scan were estimated and patients were graded based on the Lund Mackay scoring system ( High L.M score>12 , Low L.M score < 12 ). After intravenous access line establishment, induction of anesthesia was performed with O2 6 lit/min, Midazolam 1-1.5 mg, Fentanyl 2-3 mic/kg, Propofol 2 mg/kg, and Cis atracurium 0.2 mg/kg. For maintenance of anesthesia, we used Oxygen 50% Nitrous Oxide 50%, propofol (50 – 75 mic/kg/min) (DONGKOOK Pharmacy co. from Korea) and remifentanil (0.1 mic/kg/min) infusion in P group and isoflurane (0.5-1%) in the second group (Primal critical care Inc. the USA).

All of the subjects were monitored with Non-Invasive Blood Pressure, Pulse Oximetry, Electrocardiography and capnograph monitoring; through the operation. Patients’ systemic blood pressure was preserved in the mean arterial pressure (MAP) of 60-70 mmHg, and for maintaining this we used beta blockers and small doses of remifentanil. Patients’ ventilation were controlled (Tidal Volume = 10cc/kg, Respiratory Rate = 12 breath/min, FiO_2_ = 50%). They were subjected to 20 degrees trendelenburg position. Field of operation was scored based on the amount of mucosal bleeding and by a visual analogue scale according to Box 1.

**Box 1.** State of Surgical Field Throughout the Operation

	Description
**0-1**	No bleeding, excellent for surgery
**2-3**	Mild bleeding, simple surgery, not stopped operation for hemostasis or suction
**4-5**	Mild bleeding, brief difficult surgery, once stopped operation for hemorrhage or suction
**6-7**	Moderate bleeding, average difficult surgery, occasionally stopped operation for hemorrhage or suction
**8-9**	Moderate to severe bleeding, very difficult surgery, multiple stop through surgery
**10**	Termination of surgery due to severe bleeding in field

Finally collected data was analyzed by statistical software (SPSS ver.16). Chi square and T-test were used for data analysis. P value less than 0.05 was considered significant.

## 4. Results

In this study, 44 patients (26 male, 18 female) candidate for FESS were recruited from 1389 to 1390 in the Amir-Al-Momenin academic hospital. They were enrolled randomly and divided into two groups. The Mean age in Isoflurane group was 34.18 ± 13.61 years and in Propofol group was 32.3 ± 15.4 years, and no statistically significant differences were seen in demographic characteristics between the two groups (Table 1). The average length of operation (minute) was 124.31 ± 24.11 minutes in Isoflurane group and 96.36 ± 28.12 minutes in Propofol group (P = 0.001). Average level of blood loss for the subjects was 223.06 ± 157.79 ml (minimum blood loss was 50ml and maximal blood loss was 600ml). 

**Figure 1.**
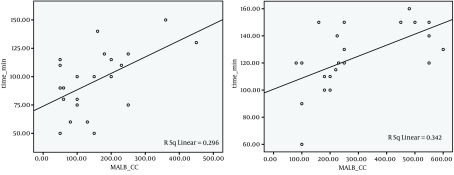
Scatter Plot Between the Amount of Intraoperative Bleeding (ml) and the Duration of Operation (minute) in Two Groups of Patients Who Used Two Kinds of Anesthesia Medicine, Propofol Group (left), Isoflurane Group (right)

**Figure 2.**
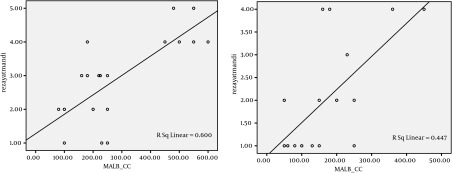
Scatter Plot Between the Amount of Intraoperative Bleeding (ml) and the Satisfaction of Surgeons During Operation in Two Groups of Patients Who Used Two Kinds Of Anesthesia Medicine Propofol Group (left), Isoflurane Group (right)

**Figure 3.**
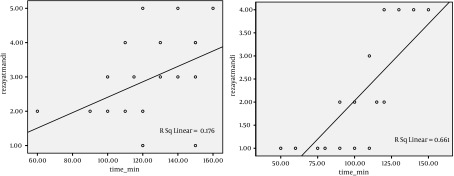
Scatter Plot Between the Duration of Operation and the Satisfaction of Surgeons During Operation in Two Groups of Patients Who Used Two Kinds of Anesthesia Medicine Propofol Group (left), Isoflurane Group (right)

The patients were divided into 3 groups according to the amount of bleeding: less than 10 percent of MABL, between 10 and 20 percent of MABL, and more than 20 percent of MABL. Amplitude of distribution of surgeon’s satisfaction ranks among two groups was meaningful (P = 0.035). Pearson correlation analysis showed that there is a positive correlation between amount of intraoperation bleeding and operation time in Isoflurane group (Pearson correlation = 0.584, P = 0.004). There was a direct correlation between operation time and amount of intraoperation bleeding. Also a positive correlation was found between the amount of intraoperative bleeding and operation time in Propofol group (Pearson correlation = 0.544, P = 0.009) (Figure 1). Spearman correlation analysis showed that there is a positive correlation between the amount of intraoperative bleeding and surgeon’s satisfaction ranks in Isoflurane group (Spearman correlation = 0.642, P = 0.001). And a positive correlation was shown in Propofol group too (Spearman correlation = 0.571, P = 0.006) (Figure 2, 3). 

**Table 1.** Frequency Distribution of Demographic Characteristics of the Study Population

Variable	Isoflurane, No. (%)	Propofol, No. (%)	Statistical Estimation
**Sex**			P = 0.76
Male	12 (54.4)	14 (63.6)	
Female	10 (45.5)	8 (36.4)	
**Responding age, y**			P = 0.807
< 20	4 (18.2)	5 (22.7)	
21-30	8 (36.4)	7 (31.8)	
31-40	4 (18.2)	6 (27.3)	
> 41	6 (27.3)	4 (18.2)	
**Age, y, Mean ± SD**	34.18 ± 13.16	32.31 ± 15.4	P = 0.673
**ASA class**			P = 0.664
Class I	18 (81.8)	20 (90.9)	
Class II	4 (18.2)	2 (9.1)	
**Lund-M**			P = 0.31
≤ 12	18 (81.8)	14 (63.6)	
> 12	4 (18.2)	8 (36.4)	

## 5. Discussion

In endoscopic sinus surgeries, establishment of controlled hypotension (MAP = 60-70 mmHg) is essential for improvement of the surgical field. This can lead to better surgical field and decrease surgical time and minimize the complications (1, 3, 4). Propofol is the most common intravenous drug, which is used in general anesthesia. Propofol provides the greatest reduction in systemic blood pressure by vasodilatation (9). Inhaled anesthetics decrease arterial blood pressure which is related to their concentration, but the mechanisms are different (10). In this study, propofol was associated with better surgical field and less amount of hemorrhage compared to Isoflurane. It may be due to reducing effect of heart rate by propofol. In a study conducted by Palvin JD et al, propofol was compared with Isoflurane and was seen to reduce the amount of bleeding in endoscopic sinus surgery (12). In another study conducted by Wonmald et al, intravenous anesthesia was associated with better results in the surgical field (15). In a study conducted by Hassani et al in Iran University of Medical Sciences, the amount of bleeding in two groups was evaluated (propofol and remifentanil versus isoflurane and remifentanil), and there was not any significant difference in the amount of bleeding between the two groups (16).

In a study conducted by Cho K et al ,propofol was compared with desflurane and was seen to reduce the intraoperative bleeding for ESS in propofol based anesthesia especially in the high –LM score patients (17) and this result was similar to our study. In another study conducted by Ahn et al., propofol was compared with sevoflurane and was seen to reduce the median blood loss especially in patients with high LM- scores (13) which was similar to our results. In our study the lower rate of bleeding was in propofol group and also surgeon’s satisfaction was more than isoflurane group. By using Numeral Logistics Regression Analysis, all of the factors were examined and it was found that surgical time (P = 0.013) was the only efficient factor on surgical bleeding. Therefore it appears that the infusion of propofol and remifentanil in comparison with isoflurane can be used in intravenous anesthesia for better vision in functional endoscopic sinus surgery.
